# Characterizing the social support and functioning of a low-threshold medication for opioid use disorder treatment cohort at intake

**DOI:** 10.1186/s12888-022-03884-5

**Published:** 2022-04-02

**Authors:** William Oles, Marcus Alexander, Navin Kumar, Benjamin Howell, Patrick G. O’Connor, Lynn M. Madden, Declan T. Barry

**Affiliations:** 1 Human Nature Lab, Yale Institute for Network Science, Yale University, New Haven, CT USA; 2 Department of Sociology, Yale University, New Haven, CT USA; 3 Section of General Internal Medicine, Yale University School of Medicine and Yale-New Haven Hospital, New Haven, CT USA; 4 VA Connecticut Healthcare System, New Haven, CT USA; 5 Section of Internal Medicine, Yale University School of Medicine and Yale-New Haven Hospital, New Haven, CT USA; 6 APT Foundation, New Haven, CT USA; 7 Department of Psychiatry, Yale University School of Medicine, New Haven, CT USA

**Keywords:** Medication for opioid use disorder, Social support, Social functioning, Low-threshold model

## Abstract

**Background:**

Despite the growing morbidity and mortality rates associated with opioid use disorder, a large gap still exists between treatment need and capacity. Low-threshold clinics utilizing medication for opioid use disorder (MOUD) treatment can increase treatment access but are understudied, and little is known about how patient demographic characteristics are associated with their social support and functioning in these settings.

**Methods:**

We used multivariate regression to estimate associations between demographic characteristics and self-reported social support or functioning indicators among patients receiving MOUD in a low-threshold clinic using several validated instruments administered at intake: Behavior and Symptom Identification Scale, Brief Pain Inventory, and Life Events Checklist for DSM-5. Patients initiating MOUD treatment between April 1 and December 31, 2017, with complete surveys were included (*N*=582).

**Results:**

Patients were primarily male (62%), aged 34 or older (53%), non-Hispanic White (79%), separated or not married (86%), and unemployed (64%). Over 20% did not live in a house or apartment in the past month. Women were more likely to “get along” with people outside their family or in social situations and to identify their partner as their source of support. Women, non-White, and older patients were at higher risk of social functioning-disrupting events (physical/sexual assaults or experiencing chronic pain), while employment and housing were protective against exposure to these trauma-related events. However, employment and housing also decreased the odds of talking with others about substance use. The aforementioned results were obtained from multivariate logistic regression models and were significant to *p*<0.05.

**Conclusions:**

Variation in support and functioning by demographic characteristics suggests that treatment facilities may benefit from adopting strategies that take baseline disparities in support and functioning into account.

**Supplementary Information:**

The online version contains supplementary material available at 10.1186/s12888-022-03884-5.

## Background

Drug overdose deaths in the United States (US) have increased in the past two decades, with overdoses involving illicit and prescription opioids being the primary driver of the epidemic [1]. Across the 12-month period ending in January 2021, the CDC reported over 94,000 drug overdose deaths, an increase of approximately 30% compared to the previous 12-month period [2]. In the wake of disruptions to health services and social distancing that have followed the COVID-19 pandemic, rates of opioid use and overdose-related morbidity and mortality have accelerated, with the largest monthly increases in drug overdose deaths since 2015 occurring in the months following statewide lockdown responses to COVID-19 [2, 3].

Medications for opioid use disorder (MOUD)—such as methadone, buprenorphine, or buprenorphine-naloxone—curb opioid cravings and decrease overdose events [4]. MOUD treatment programs have become a key public health strategy in combatting the opioid epidemic [5, 6], particularly during the COVID-19 pandemic [7]. Despite the efficacy of MOUD, a significant gap has been estimated between treatment need and treatment capacity for opioid use disorder nationwide [8, 9]. The gap exists, in part, due to barriers to access, such as health insurance coverage, waiting lists, lack of transportation, and treatment stigma [10]. Barriers exist on the prescriber side as well, with physicians facing prior authorization requirements from insurance companies and waiver training mandates from the federal government in order to prescribe many MOUD therapies [11]. Low-threshold treatment models seek to directly address barriers to care, and therefore reduce the treatment gap, in a number of ways. In contrast to traditional treatment settings, which may have rigid requirements for admission and continuation, low-threshold treatment clinics often offer same-day treatment entry, flexible medication formulations, and endorse a harm reduction approach that does not require abstinence [12].

Social support—which can be conceptualized as emotional or tangible resources shared within social relationships including expressions of sympathy, offers of advice, and physical/financial aid [13]—is associated with good health outcomes generally and substance use disorder treatment outcomes specifically. Among people who use opioids, perceived support from family or peers is related to earlier treatment seeking and longer MOUD treatment adherence [14–16]. Engaging in networks of social support is one domain of a broader concept of social functioning—the ability to “successfully engage with life and fulfill personal roles” in society [17]. The disruption of social functioning in the settings of work, school, home, or recreational life is a DSM-5 diagnostic criterion of opioid use disorder [17, 18], and improving patients’ abilities to meet responsibilities in these settings is often a target of MOUD programs. Impairment of social functioning may not only be a consequence of long-term opioid use but a precursor, as said disruptions are also intertwined with the experience of and coping with traumatic events and stress [19]. Two such examples are physical/sexual assault and chronic pain experiences, both of which can have lasting impacts on people’s ability to meet their life’s responsibilities and engage in support networks [20, 21].

Although some studies have investigated the role of social support in MOUD treatment [14–16], fewer studies have related both positive and negative indicators of social support and functioning to patient demographic characteristics at treatment initiation, and no study has done so in a low-threshold treatment setting. While we make no prior assumptions about how patients in a low-threshold treatment setting may differ from other populations, we believe the current study adds value by extending the investigation of social support and functioning in the literature on MOUD to low-threshold treatment settings. This study utilizes validated instruments administered at MOUD initiation over the course of nine months. The aims of the study are twofold: 1) to describe the demographic characteristics of a cohort of patients receiving MOUD in a low-threshold setting, and 2) to relate the cohort’s demographic characteristics to both positive and negative indicators of social support and social functioning. This analysis will allow clinicians and other administrators in the MOUD space to gain an idea of the support needs of their patient populations, which can not only strengthen existing programs through mechanisms of quality improvement but also expand planning for the scope of future MOUD treatment programs.

## Methods

### Data source and sample

Data from 588 MOUD treatment patients was gathered through surveys administered at intake to the APT Foundation in New Haven, Connecticut (CT). APT Foundation is a private, non-profit, community-based organization that specializes in treatment of opioid use disorder and which organizes a low-threshold program by walk-in admission, having locations accessible to public transportation, and treating those underinsured or uninsured [22]. Of the 588 patients, 582 had complete survey responses, with one record missing ethnicity, two records missing marital status, and three records missing employment status after data processing. Patients using MOUD at APT Foundation in New Haven were asked to complete a set of validated instruments at treatment initiation, including the Behavior and Symptom Identification Scale (BASIS-24), Brief Pain Inventory (BPI), and Life Events Checklist for DSM-5 (LEC-5). Records of all complete patient intakes from April 1, 2017 through December 31, 2017 were included. Survey measures were selected for analysis from each instrument by experts specializing in substance use disorders (authors BH, PGO, LMM, DTB), who made selections based on each measure’s pertinence to patient social support or social functioning. Although data was collected for each instrument in full as part of a standardized intake protocol, we chose to analyze individual survey items, rather than composite survey scores, since the composite scores on the whole were not necessarily designed to measure social support or functioning.

### Ethical considerations

The study involving a medical chart review was approved by the APT Foundation Board of Directors as well as the Human Investigations Committee at the Yale School of Medicine. All participants provided informed consent for data collection. All methods were performed in accordance with the relevant guidelines and regulations set forth by the APT Foundation Board of Directors and the Human Investigations Committee at the Yale School of Medicine.

### Survey instruments and key variables

BASIS-24 is a measure of self-reported psychiatric distress and functioning and can be used as a metric of mental health treatment outcomes [23]. The instrument has been demonstrated to be reliable (internal consistency *α* values ranged from 0.75 to 0.91) and discriminately valid in clinical populations [24]. Responses were drawn from three of the six survey domains (Depression and functioning, Interpersonal relationships, and Substance use). We also drew on BASIS-24 responses to abstract demographic information, including sex, age, race, ethnicity, marital status, employment status, and past-month residential status. For the purposes of analysis, we recoded sex to female/not female, race to non-White/White, ethnicity to Hispanic/non-Hispanic, marital status to married/not married, employment status to unemployed/part-time/full-time, and residential status to lived in house or apartment/did not live in house or apartment. The decision to encode demographic information in this way was made to reduce the number of comparisons and ensure model convergence given the unbalanced distribution of responses across the study cohort (Table 1). Wording for the original questions and answer choices is summarized in Supplemental Table 1 (see Additional file 1).

**Table 1:** APT Foundation 2017 cohort demographic characteristics (N=582 complete survey).

	Overall, ***% (N)***
**Sex**	
Female	38.1 (222)
Male	61.9 (360)
**Age in years**, *Mean (SD)*	36.8 (10.5)
**Race**	
White	79.0 (460)
Non-White	21.0 (122)
**Ethnicity**	
Non-Hispanic	87.5 (509)
Hispanic	12.5 (73)
**Marital status**	
Not married	85.7 (499)
Married	14.3 (83)
**Employment status**	
Unemployed	64.0 (372)
Part-time	16.1 (94)
Full-time	19.9 (116)
**Residential status**	
Didn’t live in house/apartment past 30 days	22.0 (128)
Lived in house/apartment past 30 days	78.0 (454)

In addition to individual demographic characteristics, eight survey questions were chosen for analysis from BASIS-24 which are also summarized in Supplemental Table 1 (see Additional file 1). Each item is based on the past week of the respondent’s life and includes questions about the amount of time the respondent got along with family, got along with those outside their family, and got along in social situations, all of which were considered positive indicators of social functioning and were rated on a 5-item Likert scale ranging from “None of the time” to “All of the time”. It is worth noting that labels of ‘positive’ and ‘negative’ social support or functioning were assigned by experts who specialize in substance use disorders and are familiar with survey instrument structure (authors BH, PGO, LMM, DTB). These labels are meant to help conceptualize the expected valence of each measure. For example, reporting getting along with one’s family suggests a higher quality social support or functioning in that domain per it’s inclusion in the ‘Interpersonal relationships’ BASIS-24 subscale and is therefore considered a positive indicator in the current study.

Additionally, we chose to analyze questions about how often the respondent felt close to another person, felt that they had someone to turn to for help, tried to hide their drinking or drug use, and how often others talked to them about their drinking or drug use, which were rated on a 5-item Likert scale ranging from “Never” to “Always”. Participants also indicated from a list which relationship was their main source of social support, which was recoded into three binary variables: main source of support is a partner, main source of support is family, or main source of support is a friend. The question about how often respondents tried to hide their drinking or drug use was reverse coded and interpreted as how often they did not hide their use from others, and these set of questions were considered positive indicators of social support. It should be noted, however, that being talked to by others about drinking of drug use is not always a positive indicator of support and that the impact of being talked to about those matters is often context- and relationship-dependent.

The BPI was designed to assess the severity of physical pain and the impact of pain on daily functioning [25]. The instrument has been used in a number of clinical settings and is shown to be valid and reliable (internal consistency *α* values ranged from 0.83 to 0.96) [26]. We assessed a single measure, rated 0–10, which asked how often pain interfered with the respondents’ relationships with people in the past week as a negative indicator of social functioning.

The LEC-5 was developed at the National Center for PTSD in tandem with the Clinician Administered PTSD Scale (CAPS) to assess exposure to potentially traumatic events [27], and it has been shown to be both valid and reliable as a measure of trauma history (intraclass correlation coefficients ranged from 0.62 to 0.64 for events experienced) [28]. For each traumatic event, patients indicated their experience on a nominal scale, which we recoded as a binary variable indicating whether the respondent reported that the event “Happened to me” (Supplemental Table 1, see Additional file 1). We selected five items from the LEC-5 that are negative predictors of social functioning: physical assault, assault with weapon, sexual assault, unwanted sexual experience, and the sudden and unexpected death of someone close.

### Data analysis

The objectives of our analysis were to summarize patient characteristics and to identify which characteristics are associated with reports of social support and functioning. Descriptive statistics (means, standard deviations, proportions) were calculated to examine the cohort’s demographic characteristics. For social support or functioning measures that are reported as a frequency, we employed multivariate ordinal logistic regression, which allowed us to model the log odds of each change in the response scale as a linear combination of key demographic characteristics (Supplemental Table 3, see Additional file 2). For social support or functioning measures about support relationships or experiencing a traumatic event, we employed multivariate binomial logistic regression, which models the log odds of the binary relationship or event with a linear combination of covariates (Supplemental Table 4, see Additional file 2). Results are reported as odds ratios to ease interpretability. Each model included the following covariates, which were selected by our panel of experts specializing in substance use disorders based on the availability of information gathered in the intake survey (authors BH, PGO, LMM, DTB): sex, age, race, ethnicity, marital status, employment status, and residential status. Six participant records (1%) were excluded after data cleaning for the purposes of our analysis due to missing demographic data. Missingness in indicators of social support and functioning varied but was less than 1% for any given indicator (Supplemental Table 1, see Additional file 1). Each model was compared to a null with no covariates to assess improved goodness of fit in predictions, which was confirmed by significant reduction in deviance for all models (Supplemental Tables 3 and 4, see Additional file 2). Statistical significance was set at *p*<0.05. Statistical analyses were performed using R version 3.5.2.

## Results

### Group characteristics

**Fig. 1:**
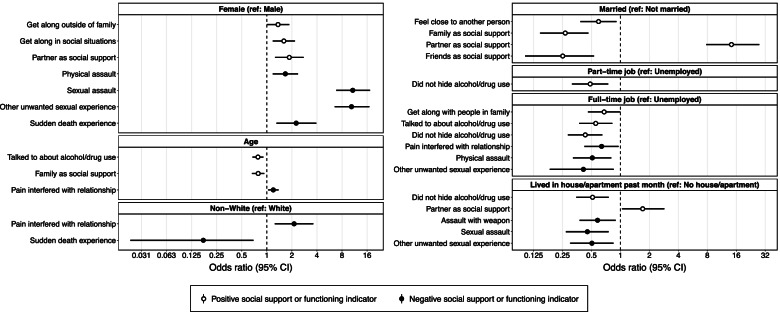
Significant adjusted odds ratios of indicators of social support and functioning by demographic characteristics. Note: Age scaled up by a factor of 10

Of 588 patients entering a low-threshold MOUD treatment program, 582 had complete baseline surveys and were considered for analysis. 222 (38%) were female, 460 (79%) were White, 73 (13%) were Hispanic, and the average age was 36.8 (SD=10.5). Additionally, 499 patients were not married (86%), 372 were unemployed (64%), and 128 (22%) reported not residing in a house or apartment in the past month. The demographic characteristics for the sample are summarized in Table 1. Significant adjusted odds ratios showing changes in probability of both positive and negative indicators of social support and functioning by demographic characteristics are summarized in Fig. 1. All results are reported in Supplemental Tables 3 and 4 (see Additional file 2).

### Positive social support and social functioning indicators

Patients with full-time jobs were 32% less likely than those unemployed to report getting along with their family (adjusted odds ratio [aOR]=0.68, 95% confidence interval [CI]=0.46–0.997), and women were 36% more likely than men to report getting along with people outside their family (aOR=1.36, 95% CI=1.002–1.86) and 60% more likely to report getting along in social situations (aOR=1.60, 95% CI=1.18–2.18). Married patients were 41% less likely to report feeling close to another person compared to non-married patients (aOR=0.59, 95% CI=0.38–0.91).

Concerning employment and age, those with full-time jobs were 45% less likely to report being talked to about their alcohol or drug use (aOR=0.55, 95% CI=0.37–0.82), and each additional 10 years in a patient’s age was associated with a 22% decrease in the likelihood that they reported being talked to about their alcohol or drug use by others (aOR=0.78, 95% CI=0.67–0.91). Being employed was associated with at least a 51% decrease in the likelihood that a patient reported not hiding their drug or alcohol use from others (Part-time employment: aOR=0.49, 95% CI=0.31–0.75; Full-time employment: aOR=0.43, 95% CI=0.28–0.65), while living in a house or apartment was associated with 49% decrease in the likelihood that the patient reported not hiding their use from others (aOR=0.51, 95% CI=0.35–0.76).

Patients also provided information about their primary source of social support. Women (aOR=1.86, 95% CI=1.26–2.77), those that lived in a house or apartment (aOR=1.70, 95% CI=1.04–2.86), and those that were married (aOR=14.25, 95% CI=7.80–27.71) were about 1.9, 1.7, and 14.3 times more likely to list their partner as their source of support, respectively. Each additional 10 years in a patient’s age was associated with a 21% decrease in the probability they reported having family as their primary support (aOR=0.79, 95% CI=0.66–0.93).

### Negative social support and social functioning indicators

Women were about 67% more likely than men to report experiencing physical assault (aOR=1.67, 95% CI=1.18–2.37) and over 10 times as likely to report sexual assault (aOR=10.76, 95% CI=6.81–17.37) or another unwanted sexual experience (aOR=10.40, 95% CI=6.53–17.06). Women were also 2.3 times more likely than men to have experienced the sudden death of someone they knew (aOR=2.25, 95% CI=1.31–3.93), while non-White patients were 83% less likely than White patients to have experienced the sudden death of another (aOR=0.17, 95% CI=0.02–0.69). Non-White patients were about 2.1 times more likely than White patients to report that they felt their experiences of pain interfered with their social relationships (aOR=2.13, 95% CI=1.25–3.62), while those employed full-time were 36% less likely to report pain interference (aOR=0.64, 95% CI=0.42–0.96). Each additional 10 years in age was associated with a 20% increase in likelihood of pain interfering with social relationships (aOR=1.20, 95% CI=1.04–1.39).

Living in a house or apartment was associated with a 55% decrease in the likelihood of experiencing sexual assault (aOR=0.45, 95% CI=0.27–0.75) and a 49% decrease in the likelihood of having an unwanted sexual experience (aOR=0.51, 95% CI=0.30–0.85). Those that resided in a house or apartment were also 42% less likely to report being assaulted with a weapon than those that did not (aOR=0.58, 95% CI=0.38–0.89). Having a full-time job was associated with a 59% decrease in the probability of having an unwanted sexual experience (aOR=0.41, 95% CI=0.18–0.85). Those with a full-time job were 49% less likely than those unemployed to report experiencing physical assault (aOR=0.51, 95% CI=0.32–0.80).

## Discussion

This study extends the current literature by identifying associations between demographic characteristics and positive and negative indicators of social support and functioning among patients entering a low-threshold MOUD program. The probability of reporting trauma experiences, social functioning disruptions, or poor network support varied across the demographic subgroups.

### Sex

Sixty-two percent of our cohort was male. We noted differences by sex in self-reported social support and functioning. Women were more likely than men to report getting along with people outside their family or in social situations and were also more likely to identify their partner as their primary source of support, despite similar rates of marriage between men and women. This may indicate that men initiating MOUD treatment are less likely to be engaged with a social support network. Women are more likely than men to rely on social support to manage problems and emotional stress, and they are also more likely to have a larger selection of drug-free network support when receiving MOUD treatment [29]. However, severe loneliness has been associated with higher odds of illicit opioid use among women in MOUD treatment programs while the same effect was not observed for men, suggesting that lack of social support may have a disproportionate impact [30]. Additionally, our results are consistent with a more general finding that women experiencing OUD are more likely to have experienced past physical, sexual, or emotional traumas [31]. More work is needed to link the differences by sex in social support and functioning to potential disparities in MOUD treatment outcomes and to understand how MOUD treatment providers might leverage this evidence to tailor support network and trauma-informed care programming to their patient populations.

### Age

The mean age of our cohort was 36.8 years and about 13% were over the age of 50, which is a smaller proportion of older patients than other traditional and low-threshold MOUD programs [32, 33]. Although older patients are typically insured and have similar if not better treatment outcomes as younger patients, older adults who use opioids are less likely to perceive their drug use as problematic and initiate treatment [31, 34]. Additionally, older patients in low-threshold MOUD programs may have different treatment needs than both older patients in other settings and younger patients in low-threshold programs.

We found that as the age of patients increased, the probability of someone talking to them about substance use or having family as primary social support decreased, while the probability of pain interfering in their relationships increased. Our findings regarding conversations about substance use and family as social support are consistent with broader epidemiological data showing a higher prevalence of social isolation among older adults relative to younger adults [35]. Given the surge in older adults seeking treatment for OUD in the United States [34], future work might focus on connecting the impact of social isolation among older adults to MOUD treatment outcomes and identifying the specific challenges imposed by isolation such as accessing healthcare for both diagnosis and treatment and maintaining emotional support throughout the treatment timeline. MOUD treatment providers might consider incorporating strategies to build support networks for patients experiencing social isolation and to address potentially modifiable factors precipitating isolation, such as living arrangement and social participation [35]. Finally, our results also highlight the need for future work to address the role of chronic pain management among older patient populations.

### Race and ethnicity

The majority of our cohort was White and non-Hispanic; however, the proportion of patients who self-identified as Hispanic (13%) was greater than other traditional and low-threshold MOUD programs [32, 33]. However, no social support or functioning indicators examined in the current study varied significantly by Hispanic ethnicity, suggesting parity between those identifying as Hispanic and non-Hispanic in measures of social support and functioning when adjusting for other demographic characteristics.

Compared to White patients, non-White patients were more likely to report that pain interfered with their relationships but less likely to have experienced the sudden death of someone they knew. The finding of disparity in pain interference is consistent with recent studies of patients experiencing chronic pain, which found that Black patients were significantly more likely than White patients to report pain interference on the BPI and that this disparity was moderated by perceived social status [36]. Racial disparities in the experience of sudden death in this setting have not been studied. Our finding that non-White patients were less likely to have experienced the sudden death of someone they knew may be explained by the greater rates of opioid-related mortality among White individuals compared to non-White individuals [37]. However, this result should also be interpreted with caution. Socioeconomic status has been shown to mediate the effects of race on probability of mortality, and since the time of data collection for this study, racial disparities in mortality have widened parallel to the progression of the COVID-19 pandemic [37, 38]. Black individuals experiencing opioid use disorder are less likely to be initiated on MOUD treatment compared to White individuals [39], and among those receiving MOUD treatment, Black individuals have lower odds of being retained in treatment for at least one year or completing treatment [40, 41]. These racial disparities in MOUD treatment are driven by structural racism and systemic inequities which lead to differential diagnosis and treatment of non-White patients, so we interpret our finding of differences in pain interference with relationships in the context of other structurally-borne barriers to treatment success for non-White patients. Future work might further investigate how racial disparities in social support or functioning are related to treatment outcomes and how structural barriers to care can be addressed in the MOUD treatment setting.

### Marital status

A small minority of our cohort was married. We found that married patients were more likely to indicate their partner as their primary source of support, but less likely to report feeling close to another person. While no other study has investigated differences in perceived closeness to others among a population of married patients receiving MOUD, recent work using a sample of heterosexual couples has shown that relationship quality is negatively correlated with MOUD-treatment-related stress for both men and women, and that women’s positive family and friend ties outside of the partnership are linked to lower stress for both partners [42]. Other work has also indicated that marriage decreases the odds of patients using cocaine or heroin during treatment and that the effect is strengthened if there is a high level of perceived closeness to the partner in the marriage [43]. This association has provided a basis for the development of behavioral couples therapy interventions in substance use disorder treatment settings, many of which have shown promise with respect to treatment outcomes [44]. Our results indicate that partnerships play an important role in perceived support and that interventions framed around increasing trust, understanding, and support among couples might further improve treatment outcomes.

### Employment status

The majority of our cohort was unemployed. A greater proportion of women were unemployed relative to men. Both full- and part-time employment were associated with hiding one’s drinking or drug use from others, while full-time employment was also associated with not getting along with one’s family and not being talked to by others about substance use. These associations suggest that holding a job may disincentivize drug use disclosure, possibly due to stigma or fear of retribution by employers or because there are fewer opportunities to talk with others due to time spent at work.

However, full-time employment was also negatively associated with reports of experiencing physical assault or unwanted sexual experiences. Since employment is a source of income and structure, it is often a target outcome of MOUD treatment programs as a way to bolster social functioning and has been associated with improved outcomes such as heroin abstinence and longer retention in treatment [41, 45, 46]. Consequently, assisting patients with obtaining employment is a potentially important target for MOUD treatment programs [47]. While our study provides no evidence for causality, the finding that employment was associated with not getting along with one’s family and hiding substance use from others warrants further consideration by those developing employment-based interventions. Additionally, existing literature has primarily focused on linking patient employment with MOUD treatment outcomes, with less work exploring how employment is related to measures of social support or functioning among patients using MOUD. Future work may provide further support for the efficacy of employment as a target outcome by investigating the psychosocial causes and consequences of employment among individuals using MOUD.

### Residential status

Twenty-two percent of our cohort reported not living in a house or apartment in the past month, which represents a greater proportion than some other traditional and low-threshold MOUD programs [32, 33]. Additionally, of the remainder of our cohort who reported living in a house or apartment, nearly 7% reported living in multiple locations in the past month, suggesting that the prevalence of unstable housing may be underestimated. People experiencing transient housing or chronic homelessness are among the most socially disenfranchised groups experiencing OUD and are therefore a target of low-threshold treatment models. Patients receiving MOUD who resided in a house or apartment in the past month were more likely to report having a partner as a source of social support and less likely to report not hiding their substance use from others, ever be assaulted with a weapon, ever be sexually assaulted, or ever have another unwanted sexual experience. Our findings suggest that those experiencing past-month housing instability seem less likely to have a support network and more likely to have experienced physical and sexual trauma. These results corroborate a larger literature relating the lack of social support and unstable housing to drug use and psychiatric comorbidities [48]. Accessible MOUD treatment approaches tailored to this subgroup are needed and have the potential to improve outcomes by increasing treatment access and providing opportunities to build drug-free networks [48, 49].

### Limitations

Our study has several limitations. Data for this study comes from surveys administered as part of a standardized intake process rather than for the investigation of our research question. Survey measures were then chosen retrospectively for analysis based on their relevance to this study. Future studies may utilize a broader range of validated instruments. The survey items chosen for this study were obtained from validated instruments that each have a unique composite scoring system; however, we chose to analyze survey items individually outside of the context of their parent instruments’ composite scores. Our analysis was cross-sectional, and further work is needed to determine causal relationships. We assessed associations between social support, functioning, and various demographic factors but did not test the association between such factors and treatment outcomes. Additionally, while our finding of differences in social support and functioning by demographic characteristics may have implications for ongoing trends in opioid use and overdose during the COVID-19 pandemic, data for this study was collected in 2017 and should therefore be interpreted in this context. Future work might also use an intersectional approach to investigate systematically the interactions between demographic characteristics and social support and functioning among patients receiving MOUD. Finally, although we attempt to adjust for a variety of demographic characteristics, the majority of our cohort was non-Hispanic White and represents a single clinic. Therefore, our findings should be read in-line with these generalizability limitations.

## Conclusions

Our low-threshold MOUD cohort represented demographic subgroups that traditionally experience social disenfranchisement, such as those with past-month housing instability. Even when controlling for other demographic covariates, both positive and negative indicators of social support and functioning varied significantly by sex, age, race, marital status, employment, and residential status. Variation in support and functioning by demographic characteristics suggests that MOUD treatment programs may benefit from adopting strategies which take baseline disparities in support and functioning into account and generate desired treatment outcomes by aligning therapies and support resources offered with their patient populations. Future work may benefit from linking measures of social support and functioning among patients receiving MOUD to treatment outcomes to gain a more complete picture of how each is related to the treatment timeline.

## Supplementary Information

**Additional file 1: Supplemental Table 1.** Demographic and social support/functioning items chosen from validated instruments. **Supplemental Table 2. **APT Foundation 2017 cohort demographic characteristics, all categories listed on BASIS-24 included with test statistics for comparison by sex.

**Additional file 2: Supplemental Table 3. **Multivariate ordinal analysis results of social support and social functioning indicators by demographic characteristics of patients entering low-threshold MOUD treatment. **Supplemental Table 4.** Multivariate binomial analysis results of social support and social functioning indicators by demographic characteristics of patients entering low-threshold MOUD treatment.

## Data Availability

The datasets generated during and analyzed during the current study are not publicly available due to limitations of ethical approval involving patient data but are available from the corresponding author (MA) on reasonable request.

## Abbreviations

MOUD: Medication for opioid use disorder
US: United States
CT: Connecticut
BASIS-24: Behavior and Symptom Identification Scale
BPI: Brief Pain Inventory
LEC-5: Life Events Checklist for DSM-5
CAPS: Clinician Administered PTSD Scale
