# Supplementary material for: Characterizing the social support and functioning of a low-threshold medication for opioid use disorder treatment cohort at intake
Source: BMC Psychiatry. 2022 Apr 2;22:236. doi: 10.1186/s12888-022-03884-5 (PMC8976510; doi:10.1186/s12888-022-03884-5)
Supplement: Supplementary file 1 — Additional file 1: Supplemental Table 1. Demographic and social support/functioning items chosen from validated instruments. Supplemental Table 2. APT Foundation 2017 cohort demographic characteristics, all categories listed on BASIS-24 included with test statistics for comparison by sex. [file 12888_2022_3884_MOESM1_ESM.docx]

**Supplemental Table 1**. Demographic and social support/functioning items chosen from validated instruments.

| **Item** | **Answer choices** | **Instrument** | **Complete (N=588)** |
| --- | --- | --- | --- |
| *Demographic items* | | | |
| How old are you? |  | BASIS-24*^a^* | 588 |
| What is your sex? | Male, Female | BASIS-24 | 588 |
| Are you… | Hispanic or Latino, not Hispanic or Latino | BASIS-24 | 587 |
| What is your racial background? | American Indian or Alaskan Native, Asian, Black or African-American, White/Caucasian, Native Hawaiian or other Pacific Islander, Multiracial or other (specify) | BASIS-24 | 588 |
| Are you now… | Married, Separated, Divorced, Widowed, Never married | BASIS-24 | 586 |
| At any time in the past 30 days, did you work at a paying job? | No, Yes 1-10 hours per week, Yes 11-30 hours per week, Yes more than 30 hours per week | BASIS-24 | 585 |
| Where did you sleep in the past 30 days? | Apartment or house, Halfway house/group home/board and care home/residential center/supervised housing, School or dormitory, Hospital or detox center, Nursing home/assisted living, Shelter/street, Jail/prison, Other (fill in) | BASIS-24 | 588 |
| *Social support and social functioning items* | | | |
| During the past week, how much of the time did you get along with people in your family? | None of the time, A little of the time, Half of the time, Most of the time, All of the time | BASIS-24 | 587 |
| During the past week, how much of the time did you get along with people outside your family? | None of the time, A little of the time, Half of the time, Most of the time, All of the time | BASIS-24 | 587 |
| During the past week, how much of the time did you get along well in social situations? | None of the time, A little of the time, Half of the time, Most of the time, All of the time | BASIS-24 | 587 |
| During the past week, how much of the time did you feel close to another person? | None of the time, A little of the time, Half of the time, Most of the time, All of the time | BASIS-24 | 587 |
| During the past week, how much of the time did you feel like you had someone to turn to if you needed help? | None of the time, A little of the time, Half of the time, Most of the time, All of the time | BASIS-24 | 588 |
| During the past week, how often did anyone talk to you about your drinking or drug use? | Never, Rarely, Sometimes, Often, Always | BASIS-24 | 582 |
| During the past week, how often did you try to hide your drinking or drug use? | Never, Rarely, Sometimes, Often, Always | BASIS-24 | 585 |
| Outside of your treatment providers, who is your main source of social support? | Wife/husband/partner, Other family (parents, children, relatives), Friends/roommates, Community/church, Other, No one | BASIS-24 | 588 |
| Circle the one number that describes how, during the past 24 hours, pain has interfered with your relationships with other people? | 0 (Does not interfere)–10 (completely interferes) | BPI*^b^* | 584 |
| Physical assault (for example, being attacked, hit, slapped, kicked, beaten up) | Happened to me, Witnessed it, Learned about it, Not sure, Doesn’t apply | LEC-5*^c^* | 588 |
| Assault with a weapon (for example, being shot, stabbed, threatened with a knife, gun, bomb) | Happened to me, Witnessed it, Learned about it, Not sure, Doesn’t apply | LEC-5 | 588 |
| Sexual assault (rape, attempted rape, made to perform any type of sexual act through force or threat of harm) | Happened to me, Witnessed it, Learned about it, Not sure, Doesn’t apply | LEC-5 | 588 |
| Other unwanted or uncomfortable sexual experience | Happened to me, Witnessed it, Learned about it, Not sure, Doesn’t apply | LEC-5 | 588 |
| Sudden, unexpected death of someone close to you | Happened to me, Witnessed it, Learned about it, Not sure, Doesn’t apply | LEC-5 | 588 |

*^a^*Behavior and Symptom Identification Scale

*^b^*Brief Pain Inventory

*^c^*Life Events Checklist for DSM-5

**Supplemental Table 2**. APT Foundation 2017 cohort demographic characteristics (N=582 complete survey), all categories listed on BASIS-24 included with test statistics for comparison by sex.

|  | | **Overall,** *% (N)* | **Female,** *%* | **Male,** *%* |  |
| --- | --- | --- | --- | --- | --- |
|  | |  |  |  |  |
| **Sex**, *% (N)* | |  | 38.1 (222) | 61.9 (360) |  |
| **Age in years**, *Mean (SD)* | | 36.8 (10.5) | 35.6 (10.5) | 37.5 (10.5) | *t*(580)=2.09, *p*=0.037 |
| **Race** | |  |  |  |  |
|  | American Indian or Alaskan native | 1.2 (7) | 0.9 | 1.4 | *X^2^*(3, *N*=582)=1.06, *p*=0.79 |
|  | Asian | 0.0 (0) | 0.0 | 0.0 |  |
|  | Black or African-American | 6.2 (36) | 5.4 | 6.7 |  |
|  | White/Caucasian | 79.0 (460) | 81.1 | 77.8 |  |
|  | Native Hawaiian or other Pacific Islander | 0.0 (0) | 0.0 | 0.0 |  |
|  | Multiracial or other | 13.6 (79) | 12.6 | 14.2 |  |
| **Ethnicity** | |  |  |  |  |
|  | Not Hispanic or Latino | 87.5 (509) | 88.3 | 86.9 | *X^2^*(1, *N*=582)=0.23, *p*=0.63 |
|  | Hispanic or Latino | 12.5 (73) | 11.7 | 13.1 |  |
| **Marital status** | |  |  |  |  |
|  | Married | 14.3 (83) | 12.6 | 15.3 | *X^2^*(4, *N*=582)=10.78, *p*=0.029 |
|  | Separated | 5.7 (33) | 7.2 | 4.7 |  |
|  | Divorced | 15.6 (91) | 17.6 | 14.4 |  |
|  | Widowed | 2.1 (12) | 4.1 | 0.8 |  |
|  | Never married | 62.4 (363) | 58.6 | 64.7 |  |
| **Employment status** | |  |  |  |  |
|  | Unemployed | 63.9 (372) | 72.5 | 58.6 | *X^2^*(3, *N*=582)=16.36, *p*<0.001 |
|  | Part-time (1-10 hours per week) | 5.5 (32) | 5.4 | 5.6 |  |
|  | Part-time (11-30 hours per week) | 10.7 (62) | 10.4 | 10.8 |  |
|  | Full-time (>30 hours per week) | 19.9 (116) | 11.7 | 25.0 |  |
| **Residential status** | |  |  |  |  |
|  | Apartment or house | 72.6 (424) | 69.4 | 75.0 | *X^2^*(7, *N*=582)=8.55, *p*=0.29 |
|  | Halfway house/group home/board and care home/residential center/supervised housing | 4.8 (28) | 4.5 | 5.0 |  |
|  | School or dormitory | 0.0 (0) | 0.0 | 0.0 |  |
|  | Hospital or detox center | 1.4 (8) | 2.3 | 0.8 |  |
|  | Nursing home/assisted living | 0.3 (2) | 0.0 | 0.6 |  |
|  | Shelter/street | 5.2 (30) | 4.5 | 5.6 |  |
|  | Jail/prison | 1.9 (11) | 1.8 | 1.9 |  |
|  | Other | 6.0 (35) | 8.1 | 4.7 |  |
|  | Multiple locations | 7.6 (44) | 9.5 | 6.4 |  |

P-value obtained from two-sample t-test for difference in distribution of age by sex, p-value obtained from chi-square test without continuity correction for associations between sex and race, ethnicity, marital status, employment status, and housing status. All tests performed using the ‘stats’ package in R, version 3.5.2.
