# Supplementary material for: Characterizing the social support and functioning of a low-threshold medication for opioid use disorder treatment cohort at intake
Source: BMC Psychiatry. 2022 Apr 2;22:236. doi: 10.1186/s12888-022-03884-5 (PMC8976510; doi:10.1186/s12888-022-03884-5)
Supplement: Supplementary file 2 — Additional file 2: Supplemental Table 3. Multivariate ordinal analysis results of social support and social functioning indicators by demographic characteristics of patients entering low-threshold MOUD treatment. Supplemental Table 4. Multivariate binomial analysis results of social support and social functioning indicators by demographic characteristics of patients entering low-threshold MOUD treatment. [file 12888_2022_3884_MOESM2_ESM.docx]

**Supplemental Table 3.** Multivariate ordinal analysis results of social support and social functioning indicators by demographic characteristics of patients entering low-threshold MOUD treatment.

|  | Get along with people in family | Get along outside of family | Get along in social situations | Feel close to another person | Have someone to turn to | Talked to about alcohol/  drug use | Did not hide alcohol/  drug use | Pain interfered with relationship |
| --- | --- | --- | --- | --- | --- | --- | --- | --- |
|  |  |  |  |  |  |  |  |  |
| Female | 1.159  [0.854–1.572]  (p = 0.34) | **1.364***  **[1.002–1.859]**  **(p = 0.049)** | **1.602****  **[1.180–2.178]**  **(p = 0.003)** | 0.799  [0.590–1.081]  (p = 0.15) | 1.045  [0.774–1.411]  (p = 0.76) | 1.317  [0.964–1.802]  (p = 0.09) | 0.910  [0.659–1.255]  (p = 0.57) | 1.187  [0.870–1.618]  (p = 0.28) |
| Age*^a^* | 0.890  [0.767–1.031]  (p = 0.12) | 1.012  [0.875–1.170]  (p = 0.87) | 0.981  [0.847–1.136]  (p = 0.80) | 1.054  [0.914–1.216]  (p = 0.47) | 1.036  [0.899–1.195]  (p = 0.62) | **0.783****  **[0.673–0.911]**  **(p = 0.002)** | 0.952  [0.817–1.110]  (p = 0.53) | **1.197***  **[1.036–1.385]**  **(p = 0.02)** |
| Non-White | 0.717  [0.424–1.207]  (p = 0.21) | 0.870  [0.515–1.463]  (p = 0.60) | 0.887  [0.522–1.497]  (p = 0.65) | 0.860  [0.514–1.440]  (p = 0.57) | 1.034  [0.615–1.737]  (p = 0.90) | 1.247  [0.720–2.168]  (p = 0.43) | 0.572  [0.325–1.000]  (p = 0.05) | **2.128****  **[1.246–3.621]**  **(p = 0.006)** |
| Hispanic | 1.227  [0.653–2.307]  (p = 0.53) | 1.086  [0.574–2.056]  (p = 0.80) | 1.208  [0.638–2.292]  (p = 0.56) | 0.930  [0.499–1.731]  (p = 0.82) | 0.861  [0.456–1.628]  (p = 0.65) | 1.099  [0.565–2.133]  (p = 0.78) | 1.270  [0.643–2.518]  (p = 0.49) | 0.776  [0.403–1.491]  (p = 0.45) |
| Married | 1.338  [0.874–2.053]  (p = 0.18) | 1.102  [0.715–1.695]  (p = 0.66) | 1.341  [0.874–2.056]  (p = 0.18) | **0.592***  **[0.383–0.910]**  **(p = 0.02)** | 0.805  [0.521–1.241]  (p = 0.33) | 0.942  [0.599–1.480]  (p = 0.80) | 1.234  [0.779–1.953]  (p = 0.37) | 0.724  [0.459–1.132]  (p = 0.16) |
| Part-time job | 0.882  [0.584–1.330]  (p = 0.55) | 0.921  [0.604–1.403]  (p = 0.70) | 0.999  [0.657–1.517]  (p = 1.00) | 1.010  [0.678–1.505]  (p = 0.96) | 1.412  [0.942–2.118]  (p = 0.095) | 0.757  [0.499–1.149]  (p = 0.19) | **0.485****  **[0.313–0.747]**  **(p = 0.002)** | 0.735  [0.482–1.113]  (p = 0.15) |
| Full-time job | **0.677***  **[0.459–0.997]**  **(p = 0.049)** | 0.821  [0.554–1.215]  (p = 0.33) | 0.831  [0.563–1.226]  (p = 0.35) | 0.930  [0.628–1.377]  (p = 0.72) | 0.808  [0.543–1.199]  (p = 0.29) | **0.553****  **[0.372–0.822]**  **(p = 0.004)** | **0.431*****  **[0.284–0.648]**  **(p < 0.001)** | **0.637***  **[0.421–0.957]**  **(p = 0.03)** |
| Lived in house/apartment past 30 days | 0.929  [0.643–1.342]  (p = 0.70) | 0.944  [0.652–1.368]  (p = 0.76) | 0.786  [0.544–1.135]  (p = 0.20) | 0.714  [0.495–1.030]  (p = 0.072) | 0.759  [0.528–1.091]  (p = 0.14) | 0.689  [0.472–1.006]  (p = 0.06) | **0.513****  **[0.347–0.756]**  **(p = 0.001)** | 0.988  [0.688–1.423]  (p = 0.95) |
| Have urge to drink or use drugs*^b^* |  |  |  |  |  | **1.620*****  **[1.424–1.847]**  **(p < 0.001)** | **0.445*****  **[0.384–0.514]**  **(p < 0.001)** |  |
| Observations | 581 | 581 | 581 | 581 | 582 | 574 | 577 | 578 |
| Deviance | 1814 | 1735 | 1769 | 1824 | 1795 | 1669 | 1581 | 2235 |
| Null deviance | 1844 | 1762 | 1805 | 1856 | 1825 | 1780 | 1783 | 2293 |

aOR [95% CI] (p value)

*p<0.05, **p<0.01, ***p<0.001. Bolded text indicates significance of at least p<0.05. Fit statistics show the sum of squared deviance between predicted and observed values for the model and a null model with no covariates.

*^a^*Age scaled up by 10.

*^b^*Answers to survey item “During the PAST WEEK, how often did you have an urge to drink alcohol or take street drugs” from BASIS-24 were included as a control in analysis of survey answers about talking to others about alcohol/drug use.

**Supplemental Table 4.** Multivariate binomial analysis results of social support and social functioning indicators by demographic characteristics of patients entering low-threshold MOUD treatment.

|  | Partner as social support | Family as social support | Friends as social support | Physical assault | Assault with weapon | Sexual assault | Other unwanted sexual experience | Sudden death experience |
| --- | --- | --- | --- | --- | --- | --- | --- | --- |
|  |  |  |  |  |  |  |  |  |
| Female | **1.863****  **[1.258–2.769]**  **(p = 0.002)** | 1.000  [0.703–1.423]  (p = 1.00) | 1.058  [0.701–1.588]  (p = 0.79) | **1.670****  **[1.179–2.371]**  **(p = 0.004)** | 0.928  [0.631–1.358]  (p = 0.70) | **10.763*****  **[6.811–17.374]**  **(p < 0.001)** | **10.402*****  **[6.528–17.059]**  **(p < 0.001)** | **2.254****  **[1.307–3.929]**  **(p = 0.004)** |
| Age*^a^* | 0.851  [0.699–1.031]  (p = 0.10) | **0.788****  **[0.664–0.932]**  **(p = 0.006)** | 1.002  [0.823–1.216]  (p = 0.98) | 0.858  [0.726–1.012]  (p = 0.07) | 1.119  [0.937–1.335]  (p = 0.21) | 0.967  [0.780–1.196]  (p = 0.76) | 0.884  [0.708–1.098]  (p = 0.27) | 1.115  [0.860–1.433]  (p = 0.40) |
| Non-White | 0.852  [0.402–1.733]  (p = 0.67) | 0.968  [0.516–1.801]  (p = 0.92) | 0.831  [0.370–1.720]  (p = 0.64) | 0.590  [0.312–1.091]  (p = 0.10) | 0.777  [0.388–1.481]  (p = 0.46) | 0.897  [0.387–1.981]  (p = 0.79) | 0.832  [0.348–1.877]  (p = 0.67) | **0.173***  **[0.023–0.692]**  **(p = 0.04)** |
| Hispanic | 1.306  [0.552–3.147]  (p = 0.55) | 0.869  [0.408–1.850]  (p = 0.71) | 1.079  [0.438–2.746]  (p = 0.87) | 2.010  [0.953–4.323]  (p = 0.07) | 1.666  [0.761–3.746]  (p = 0.21) | 1.023  [0.381–2.779]  (p = 0.96) | 0.862  [0.307–2.446]  (p = 0.78) | 2.999  [0.555–24.252]  (p = 0.24) |
| Married | **14.253*****  **[7.795–27.713]**  **(p < 0.001)** | **0.267*****  **[0.146–0.465]**  **(p < 0.001)** | **0.251****  **[0.102–0.531]**  **(p = 0.001)** | 0.931  [0.563–1.535]  (p = 0.78) | 1.175  [0.682–1.983]  (p = 0.55) | 0.645  [0.310–1.280]  (p = 0.22) | 0.752  [0.360–1.503]  (p = 0.43) | 1.299  [0.581–2.682]  (p = 0.50) |
| Part-time job | 0.779  [0.445–1.333]  (p = 0.37) | 0.886  [0.550–1.424]  (p = 0.62) | 0.665  [0.359–1.176]  (p = 0.18) | 0.715  [0.445–1.143]  (p = 0.16) | 0.921  [0.544–1.526]  (p = 0.75) | 1.147  [0.623–2.085]  (p = 0.66) | 1.217  [0.661–2.213]  (p = 0.52) | 0.899  [0.389–1.890]  (p = 0.79) |
| Full-time job | 1.227  [0.742–2.014]  (p = 0.42) | 0.797  [0.504–1.256]  (p = 0.33) | 1.083  [0.645–1.792]  (p = 0.76) | **0.510****  **[0.321–0.803]**  **(p = 0.004)** | 0.608  [0.351–1.020]  (p = 0.07) | 0.556  [0.268–1.095]  (p = 0.10) | **0.412***  **[0.184–0.850]**  **(p = 0.02)** | 0.892  [0.396–1.864]  (p = 0.77) |
| Lived in house/apartment past 30 days | **1.702***  **[1.038–2.862]**  **(p = 0.04)** | 1.473  [0.969–2.250]  (p = 0.07) | 1.546  [0.938–2.625]  (p = 0.10) | 0.693  [0.454–1.054]  (p = 0.09) | **0.578***  **[0.376–0.893]**  **(p = 0.01)** | **0.453****  **[0.271–0.754]**  **(p = 0.003)** | **0.505***  **[0.300–0.847]**  **(p = 0.01)** | 0.720  [0.387–1.384]  (p = 0.31) |
| Observations | 582 | 582 | 582 | 582 | 582 | 582 | 582 | 582 |
| Deviance | 641 | 763 | 610 | 772 | 680 | 501 | 485 | 375 |
| Null deviance | 753 | 805 | 631 | 807 | 696 | 651 | 631 | 395 |

aOR [95% CI] (p value)

*p<0.05, **p<0.01, ***p<0.001. Bolded text indicates significance of at least p<0.05. Fit statistics show the sum of squared deviance between predicted and observed values for the model and a null model with no covariates.

*^a^*Age scaled up by 10.
